# Implementing the GSA KAER Toolkit to Enhance Detection and Management of Cognitive Impairment in Older Adults

**DOI:** 10.1093/geroni/igaf122.4139

**Published:** 2025-12-31

**Authors:** Anna Pendrey, Triccia Aparicio, Miguel Paz, Mariel Zelaya, Javier Sevilla, Samir Cabrera, Paola Rodriguez, Omar Suazo

**Affiliations:** Indiana University, Zionsville, Indiana, United States; Universidad Nacional Autonoma de Honduras, Zionsville, Indiana, United States; Universidad Nacional Autonoma de Honduras, Zionsville, Indiana, United States; Universidad Nacional Autonoma de Honduras, Zionsville, Indiana, United States; Indiana University, Zionsville, Indiana, United States; UNITEC, Zionsville, Indiana, United States; Indiana University, Zionsville, Indiana, United States; UNITEC, Zionsville, Indiana, United States

## Abstract

Background Cognitive impairment and dementia affect older adults in the US but often go undiagnosed in primary care due to time constraints, stigma, and lack of standardized protocols. The Gerontological Society of America’s KAER (Kickstart, Assess, Evaluate, Refer) Toolkit offers a structured approach to improve early identification and management. Methods This quality improvement project was conducted in a primary care brain clinic from September 2023 to June 2025 for patients aged ≥65 years. Cognitive screening was integrated into routine visits. Patients with concerns identified in the Kickstart phase underwent detailed history-taking, functional assessment, and standardized testing, including the Montreal Cognitive Assessment and Patient Health Questionnaire-9. Reversible causes were investigated, and individualized care plans were developed with referrals to subspecialty care and community resources. Data collected included prevalence, comorbidities, functional status, and referral patterns. Results Among 41 patients screened, 80% had cognitive impairment, most aged 66–75 years. Women were more likely to have dementia; men more often had mild cognitive impairment. Patients comprised 42% African American, 27% Hispanic, and 27% White. Depression (83%) and anxiety (67%) were common, along with comorbidities such as hypertension (23%) and chronic kidney disease (23%). Two patients had positive ApoE genotypes with MRI-confirmed pathology. Conclusions KAER Toolkit implementation identified high rates of cognitive impairment and comorbidities, supporting its user-friendly design to improve early detection, targeted interventions, and coordinated care in older adults.

